# The soapberry bug, *Jadera haematoloma* (Insecta, Hemiptera, Rhopalidae): First Asian record, with a review of bionomics

**DOI:** 10.3897/zookeys.297.4695

**Published:** 2013-05-08

**Authors:** Jing-Fu Tsai, Yi-Xuan Hsieh, Dávid Rédei

**Affiliations:** 1 Insect Collection, Taiwan Forestry Research Institute, 4F, No 67, Sanyuan St., Zhongzheng Dist., Taipei City 100, Taiwan; 2 Kaohsiung Association of Naturalists, Kaohsiung, Taiwan; 3 Institute of Entomology, College of Life Sciences, Nankai University, 94 Weijin Road, Tianjin, 300071, China; 4 Department of Zoology, Hungarian Natural History Museum, H-1088 Budapest, Baross u. 13, Hungary

**Keywords:** Hemiptera, *Jadera haematoloma*, alien species, invasion, rapid evolution, Sapindaceae, Asia

## Abstract

The soapberry bug, *Jadera haematoloma* (Herrich-Schäffer, 1847) (Insecta: Hemiptera: Heteroptera: Rhopalidae: Serinethinae), a species native in tropical and subtropical regions of the New World and accidentally introduced to Hawaii, is reported for the first time from Asia (Taiwan). This record represents the first occurrence of the species in Asia. Stable populations composed of hundreds of specimens were found in seven localities of Kaohsiung City and one locality in Tainan City, and a single specimen was observed in Chiayi County. Aggregating adults and larvae fed in large numbers on the sapindacean plants *Cardiospermum halicacabum* L. and *Koelreuteria elegans* (Seem.) A. C. Smith ssp. *formosana* (Hayata) F. G. Meyer. Diagnostic characters of adults and larvae of *Jadera haematoloma* are discussed. A review of its bionomics and a bibliography are provided. Initial observations on the populations in southern Taiwan are presented. The species is potentially invasive, and further extension of its range is anticipated in Southeast Asia.

## Introduction

Soapberry bugs (Hemiptera: Heteroptera: Rhopalidae: Serinethinae) are seed predators feeding exclusively on members of the soapberry family (Sapindaceae). The subfamily contains three genera: *Leptocoris* Hahn, 1833 (more than 40 species) is found throughout the tropical and subtropical regions of the Old World (Gross 1960, Göllner-Scheiding 1980, 1982, 1983); *Jadera* Stål, 1862 (about 17 species) is restricted to the New World (Göllner-Scheiding 1979, 1982, 1983) with a single species introduced to Hawaii in the 1960s (Davis 1969, Gagné 1971a, b); and *Boisea* Kirkaldy, 1910 (4 species) has a disjunct distribution, with one species in tropical Africa, one in the Indian subcontinent, and two in North America (Göllner-Scheiding 1980, 1982, 1983).

The best-known species of *Jadera* is *Jadera haematoloma* (Herrich-Schäffer, 1847), commonly called the soapberry bug or the red shouldered bug. It is widely distributed in tropical and subtropical regions of North, Central and northern South America (Göllner-Scheiding 1979) and also found in temperate parts of the USA. It is one of the most common species of the genus *Jadera* in North America, frequently forming large aggregations on various native and cultivated soapberries (Carroll and Loye 1987). In the United States it was restricted to the southern states until the 1980s, but it began to extend its range northward in the mid-1980s (Hoffman and Steiner 2005). It was introduced to Hawaii in the 1960’s (Davis 1969, Gagné 1971a, 1971b).

A single individual of *Jadera haematoloma* was found in Dagangshan Scenic Area, Alian District, Kaohsiung City, southern Taiwan on 31 August 2012 by Y.X. Hsieh and J.X. Fang. Subsequent targeted search in the region resulted in discovery of populations at seven localities. These represent the first occurrences of this species and the genus *Jadera* in Asia. We provide the first records of *Jadera haematoloma* with data on its distribution, population and host plants in Taiwan, present the diagnostic characters allowing its recognition, document the immature stages, and provide a bibliography and a review of the bionomics, ecology, and distribution of this species.

## A review of *Jadera haematoloma*

***Jadera haematoloma* (Herrich-Schäffer, 1847)**

*Leptocoris haematoloma* Herrich-Schäffer, 1847: 103. Syntype (s): Mexico; lost? (Göllner-Scheiding 1975: 57).

*Lygaeus marginalis* Walker, 1872: 45. Lectotypus (Göllner-Scheiding 1979: 57) (female): Mexico, “Oajaca” [= Oaxaca]; deposited in the Natural History Museum, London, UK. Synonymized by Distant (1901: 540).

### Bibliography

*Serinetha haematoloma*: Dallas 1852: 463 (record), Dohrn 1859: 27 (catalogue, distribution).

*Lygaeus (Serinetha) haematolomus*: Guérin-Méneville 1857: 393 (diagnosis, record).

*Jadera haematoloma*: Stål 1862: 307 (listed), Stål 1870: 226 (listed, distribution, variability), Walker 1871: 145 (listed, distribution), Uhler 1872: 404 (distribution), Glover 1876: 43 (listed, habitus), Uhler 1876: 302 (distribution, wing polymorphism), Distant 1882: 173 (habitus, record, distribution), Provancher 1886: 65 (listed), Berg 1892: 104 (records, distribution), Distant 1893: 378 (records), Lethierry and Severin 1894: 124 (catalogue, distribution), Uhler 1894: 237 (records), Gillette and Baker 1895: 21 (listed), Distant 1901: 540 (synonymy), Sanderson 1905: 21 (host plant, economic importance), Barber 1906: 272 (record, wing polymorphism, distribution), Sanderson 1906: 47 (record, host plant, aggregation, oviposition), Snow 1906: 152 (record), Van Duzee 1909: 163 (record), Van Duzee 1916: 15 (listed), Van Duzee 1917: 127 (catalogue, distribution), Barber 1923: 23 (record, wing polymorphism, distribution), Van Duzee 1923: 136 (records), Blatchley 1926: 286 (in key, redescription, records, wing polymorphism), Deay 1928: 400 (original description translated, figures of male genitalia, records), Torre-Bueno 1930: 107 (record), Torre-Bueno 1931: 137 (record), Harris 1937: 172 (records), Brimley 1938: 65 (record), Torre-Bueno 1941: 101 (in key, distribution), Froeschner 1942: 596 (diagnosis), 604 (records, phenology), Barber and Bruner 1947: 88 (record), Sherman 1948: 17 (record), Drew and Schaefer 1963: 113 (in key), 120 (distribution, habitus, host plant), Schaefer 1965: 10 (listed, morphology, wing, abdomen, male and female genitalia, figures), Alayo 1967: 35 (in key), 36 (diagnosis, records), Chopra 1967: 365 (listed, figures of male genitalia), Davis 1969: 274 (record, distribution, host plant, aggregation), Gagné 1971a: 3 (record, host plant), Gagné 1971b: 24 (record, host plant, feeding), Hepburn and Yonke 1972 (morphology and figure of metathoracic scent gland), Schaefer 1977: 287 (genital capsule, figure), Grillo and Alayo 1978: 61 (records), 62 (in key), Schaefer 1978: 660 (listed, genital capsule), Slater and Baranowski 1978: 68 (diagnosis, habitus, distribution), Aldrich et al. 1979: 324 (host plant, laboratory rearing, chemical ecology), Göllner-Scheiding 1979: 57 (redescription, lectotype designation, genitalia, figures, distribution), Ueshima 1979: 73 (karyotype), Hoebeke and Wheeler 1982: 219 (in key, male genitalia, figure, distribution), Schaefer and Chopra 1982: 226 (morphology, host plants); Göllner-Scheiding 1983: 179 (catalogue, distribution), Schaefer and Mitchell 1983: 593 (host plants), Mead 1985: [1] (diagnostic characters, wing polymorphism, larva, photos, host plant, distribution, map, phenology, aggregation, impact on human, control), Carroll and Loye 1987: 373 (host plants, feeding, coevolution with host plants, aggregation, ecology, distribution, map), Carroll 1988: 54 (records, distribution, host plants, phenology, development, reproductive behaviour and ecology), Henry 1988: 663 (catalogue, distribution), Maes and Tellez Robleto 1988: 5, 23, 40, 58 (host plants), Ribeiro 1989: 466 (records, host plant, aggregation, aposematism, development), Aldrich et al. 1990a: 200 (laboratory rearing, chemical ecology), Aldrich et al. 1990b: 370 (records, host plants, laboratory rearing, chemical ecology), Carroll 1991: 510 (reproductive behaviour and ecology), Carroll and Boyd 1992: 1053 (records, host plants, feeding, intraspecific variability, evolution), Carroll 1993: 156 (reproductive ecology), Maes and Göllner-Scheiding 1993: 15 (listed, distribution, host plants), McLennan et al. 1994: 936 (records, colour polymorphism), Arnold 1995: 13 (record, distribution, habitat), Carroll and Corneli 1995: 47 (reproductive ecology), Carroll and Salamon 1995: 1463 (reproductive ecology), Carroll and Dingle 1996: 210 (records, host plants, feeding, intraspecific variability, evolution), Carroll et al. 1997: 1182 (genetic architecture, interspecific variability, selection), Dingle and Winchell 1997: 365 (genetic and physiological control of wing polymorphism), Carroll et al. 1998: 956 (records, host plants, reproductive ecology, adaptation), Froeschner 1999: 236 (listed), Reinert et al. 1999: 469 (pest status, biological control), Schaefer and Kotulski 2000: 312 (host plants, economic importance), Winchell et al. 2000: 1365 (wing polymorphism, physiology), Carroll et al. 2001: 258 (genetic architecture, interspecific variability, selection), Carroll et al. 2003a: S80 (genetic variation, selection), Carroll et al. 2003b: 135 (host plants, records, polymorphism), Hoffman and Steiner 2005: 7 (distribution, records, host plant), Dingle et al. 2009: 2031 (genetic architecture, intraspecific variability, selection), Zych 2010: 644 (aggregation), Carroll and Loye 2012: 675 (host plants), Zych et al. 2012 (stridulation).

*Pyrrhotes haematoloma*: Banks 1910: 73 (catalogue, distribution), Barber 1914: 518 (records), Malloch 1918: 284 (records), Blöte 1934: 269 (listed, record).

*Leptocoris haematoloma*: Porter 1917: 316 (host plant, record, spermatogenesis), Makino 1951: 134 (karyotype).

### Diagnostic characters of adult

The genus *Jadera* can be recognized within Serinethinae by the long bucculae which approach base of head posteriorly; in the two other genera of the subfamily, *Leptocoris* and *Boisea*, they are short, at most extending to middle of ventral surface of head (Schaefer 1965, Göllner-Scheiding 1979). No native Asian member of Serinethinae shares this character.

**Figures 1–2.**
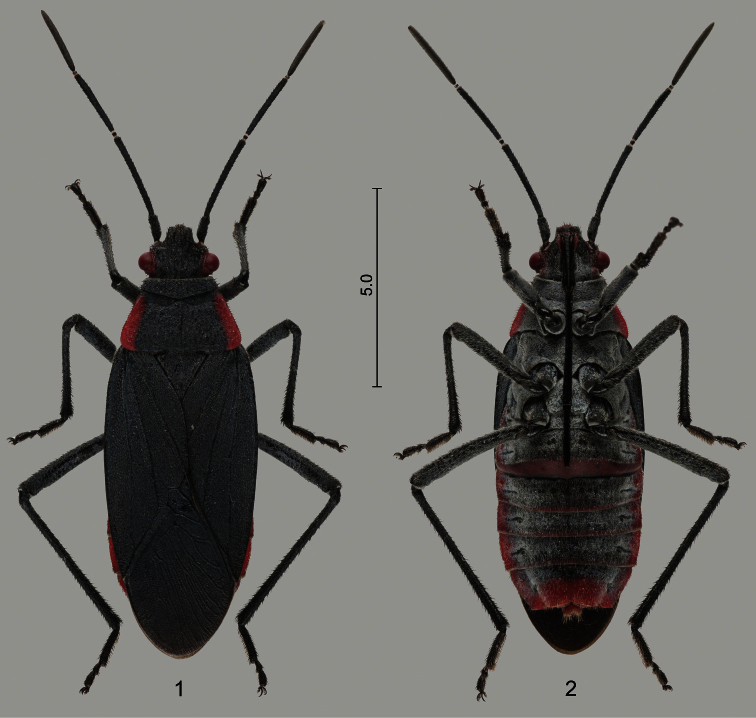
Female of *Jadera haematoloma* **1** dorsal view **2** ventral view. Scale bar in mm.

**Figures 3–5.**
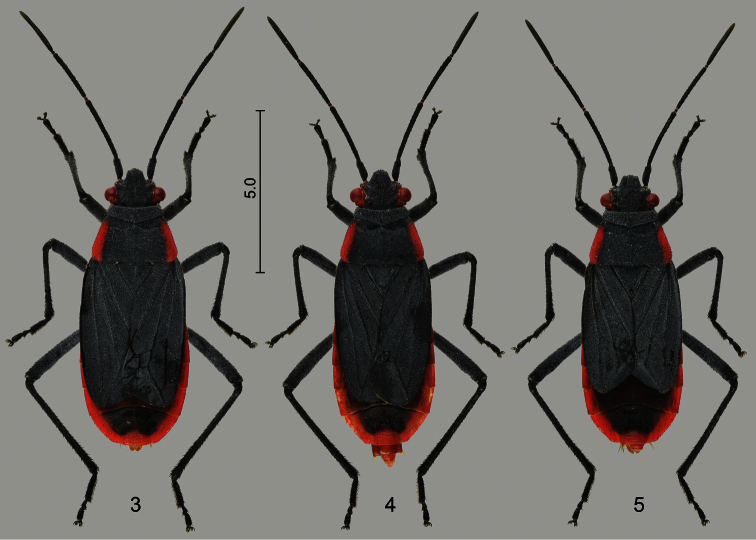
Brachypterous males of *Jadera haematoloma*, with different wing shapes, dorsal views. Scale bar in mm.

*Jadera haematoloma* is a medium-sized species within the genus (9.5–14.5 mm) readily recognized by its colour (Figs 1–5): dorsal ground colour black, head with a narrow red stripe along each eye, and pronotum broadly margined with bright red laterally; abdominal venter black, lateral margins, posterior margin of sternite VI and posterior third of sternite VII broadly red (occasionally more extensively red). Only two other species of the genus have a uniformly black dorsum with contrasting red lateral margins of the pronotum: *Jadera pyrrholoma* Stål, 1870 and *Jadera diaphona* Göllner-Scheiding, 1982. The South American *Jadera pyrrholoma* differs from *Jadera haematoloma*, among others, by its considerably greater size (14.0–18.5 mm) and its uniformly red abdomen. The Central American *Jadera diaphona* is similar to *Jadera haematoloma*, but it has a uniformly orange abdominal venter. Detailed morphological redescriptions of *Jadera haematoloma* and other congeners were provided by Göllner-Scheiding (1979); for distinguishing it from *Jadera diaphona*, the subsequent paper by Göllner-Scheiding (1982) also should be consulted. The male genitalia of the species are diagnostic; they were illustrated by Deay (1928), Schaefer (1965, 1977, 1978), Chopra (1967), Göllner-Scheiding (1979), and Hoebeke and Wheeler (1982).

### Larval instars

A brief description and photo of the fifth instar were provided by Mead (1985).

### Intraspecific variability

**Body size.** Both males and females in regions of southcentral USA (Oklahoma) are significantly smaller than those in tropical areas (southern Florida) (Carroll and Loye 1987, Carroll 1988). Macropterous morphs are also significantly larger than brachypters (Carroll et al. 2003b). In Florida, members of populations on a native host plant (*Cardiospermum corindum*) are slightly greater than those on an introduced host plant (*Koelreuteria elegans*); the difference is not significant (Carroll et al. 1997, 1998, 2001).

**Colour pattern** varies only slightly within a population. Caribbean specimens (Bahamas, Cuba) usually have broader vitta along the lateral margin of pronotum, red pattern is present on thoracic pleuron, and the apex of the clypeus also is red (Carroll and Boyd 1992). Colour variants were observed in Mexico (McLennan et al. 1994); some of them have the abdominal venter extensively red (Carroll and Boyd 1992) but this latter record needs confirmation because of possible confusion with *Jadera diaphona*. Two colour variants, ‘orange’ and ‘lemon’, were described from laboratory cultures, but they are rare in nature; inheritance of these colour morphs apparently follows a two-locus/two-allele mode, with the two loci interacting epistatically (McLennan et al. 1994).

**Wing polymorphism.** Usually macropterous (Figs 1–2); approximately 20% of the population in the southern USA is brachypterous (Carroll et al. 2003b). The brachypterous morph was illustrated by Glover (1876), Mead (1985) and Carroll et al. (2003b); such specimens have the corium and membrane shortened (Figs 3–5) and their flight muscles are lacking (Carroll et al. 2003b). Macropterous morph encompasses three states of flight muscle development: flight muscles developed and retained; flight muscles histolysed; and flight muscles congenitally lacking (Dingle and Winchell 1997, Winchell et al. 2000, Carroll 2003b). As a result, a considerable proportion (about one half in average) of the macropterous individuals is cryptic flightless in some populations.

Frequency of wing morphs is under complex genetic and physiological regulation. Crossing experiments indicate a polygenic inheritance of wing morphs (Dingle and Winchell 1997). Frequency of the flightless (brachypterous and histolyzing macropterous) morphs does not differ in populations colonizing native and introduced host plant in Florida; however, congenitally flightless macropterous specimens were more common on the introduced host plant (Carroll et al. 2003b). In populations feeding on native host plants flightless morphs have significantly lower activities of selected enzymes involved in glycolysis, oxidative metabolism and fatty acid oxidation than flyers, but there is no difference in populations feeding on introduced host plants (Winchell et al. 2000). In laboratory cultures of developing larvae from populations on native the host plant, a significant negative correlation between food level and macroptery ratio was documented: increase in available food results in a decrease in the percentage of macropterous individuals. Treatment with a juvenile hormone analog (methoprene) tends to increase the proportion of brachypterous morphs, but response of the different populations is different (Dingle and Winchell 1997). Rearing at different temperatures does not affect wing-morph frequencies (Dingle and Winchell 1997), but wing development is influenced by photoperiod (Carroll et al. 2003b).

**Length of labium.** The labium is significantly longer in macropterous specimens (Carroll et al. 2003b). Significant differences in the length of the labium correlating with fruit morphology of the host plant were documented in local populations; see ‘Coevolution with host plants’.

### Karyotype

Male diploid chromosome number is 13 (10A+2m+X0) (Porter 1917, Makino 1951, Ueshima 1979).

### Habitat

*Jadera haematoloma* colonizes various habitats where host plants are available and can be found in city parks and other human-dominated environments (Carroll and Boyd 1992). Adults and larvae usually are found in the canopy and on the trunk of host plants (Carroll and Loye 1987, Carroll 1988), but they also are found on the ground in leaf litter where they feed on fallen seeds (Gagné 1971b, Carroll 1988).

### Diet

As all other members of the subfamily Serinethinae, *Jadera haematoloma* is an oligophagous seed-predator that develops exclusively on plants of the soapberry family (Sapindaceae s. lato, including the former Hippocastanaceae and Aceraceae). All of its hosts belong to the subfamily Sapindoideae. In contrast to several other congeners, which are restricted to members of the tribe Paullinieae, *Jadera haematoloma* also feeds on plants of the subfamilies Sapindeae (*Sapindus*) and Koelreuteriae (*Koelreuteria*) (Table 1) (Schaefer and Mitchell 1983, Carroll and Loye 1987, 2012).

**Table 1.** Host plants of *Jadera haematoloma* at different localities and reports of aggregation behaviour or mass occurrence based on literature data.<br/>

**Host plant**	**Locality**	**Aggregation**	**References**
*Sapindus saponaria* L.	Hawaii		Carroll and Loye (2012)
*Sapindus saponaria* L. var. *drummondii* (Hook. & Arn.) L.D. Benson	Arizona	+	Carroll and Loye (1987), Ribeiro (1989), Aldrich et al. (1990b)
	Kansas	–	Carroll and Loye (1987)
	Oklahoma	+	Carroll and Loye (1987), Carroll and Boyd (1992)
*Sapinus oahuensis* Hillebr. ex Radlk.	Hawaii	+	Gagné (1971b)
*Sapindus mukorossi* Gaertn.	USA		Carroll and Loye (2012)
*Koelreuteria paniculata* Laxm.	Florida	+	Carroll and Loye (1987), Ribeiro (1989), Aldrich et al. (1990b)
	Georgia		Carroll and Loye (1987)
	Missouri		Carroll and Loye (1987)
	New Mexico		Carroll and Loye (1987)
	Oklahoma	+	Carroll and Loye (1987), Carroll and Boyd (1992)
*Koelreuteria elegans* (Seem.) A.C. Smith	Florida	+	Carroll and Loye (1987), Carroll and Boyd (1992), Carroll et al. (1997, 1998, 2003b)
*Koelreuteria elegans* subsp. *formosana* (Hayata) F.G. Meyer	Hawaii	+	Gagné (1971a, b)
*Koelreuteria bipinnata* Franch.	USA		Carroll and Loye (2012)
*Koelreuteria* sp. (unspecified)	North Carolina		Hoffman and Steiner (2005)
*Cardiospermum halicacabum* L.	Texas		Porter (1917)
	Mississippi		Carroll and Loye (1987)
	Louisiana		Carroll and Boyd (1992)
	Hawaii	+	Davis (1969)
	Bahamas		Carroll and Loye (2012)
*Cardiospermum corindum* L.	Florida	+	Carroll and Loye (1987, 2012), Aldrich et al. (1990b), Carroll and Boyd (1992), Carroll et al. (1997, 1998, 2003b)
	Mexico		Carroll and Loye (2012)
*Cardiospermum grandiflorum* Sw.	California		Carroll and Loye (2012)
	Hawaii		Carroll and Loye (2012)
*Serjania brachycarpa* A.Gray ex Radlk.	Texas	+	Carroll and Loye (1987), Carroll and Boyd (1992)

In the southwestern USA its primary native host plant is the western soapberry (*Sapindus saponaria* var. *drummondii*), but it also can be found in large numbers on the littlefruit slipplejack (*Serjania brachycarpa*). Within its native area it also successfully colonizes several sapindaceous trees introduced to that region, e.g. large aggregations are commonly found on the goldenrain tree (*Koelreuteria paniculata*) and the Chinese rain tree (*Koelreuteria elegans*), which are native to eastern Asia and introduced in the southern part of the United States (Carroll and Loye 1987). The heartseed vine (*Cardiospermum halicacabum*), a widely distributed subtropical climbing plant of uncertain provenance is also present to the southern part of the USA, and is frequently colonized by *Jadera haematoloma* in Louisiana and Mississippi where the tree is common, but this plant is apparently not used as a host in southern Oklahoma and northern Texas where it is less common (Carroll and Boyd 1992).

In Florida the bug is common on the native balloon vine (*Cardiospermum corindum*) and also feeds on the introduced *Sapindus mukorossii* but avoids a native congener, *Sapindus saponaria* (Carroll and Loye 2012). After it was introduced to Hawaii, *Jadera haematoloma* quickly colonized sapindaceans not occurring in its native area, some of which are native to Hawaii (*Sapinus oahuensis*); other hosts are introduced (*Koelreuteria elegans* subsp. *formosana* and *Cardiospermum grandiflorum*) (Carroll and Loye 1987, 2012).

*Jadera haematoloma* occasionally has been reported from plants belonging to other families, e.g. from *Ficus brevifolia* Nutt. and unspecified species of *Ficus* (Moraceae) (Aldrich et al. 1979, Mead 1985, Maes and Göllner-Scheiding 1993), *Althaea* sp. (Malvaceae) (Schaefer and Chopra 1982, Mead 1985, Maes and Göllner-Scheiding 1993), cassava (*Manihot esculenta* Crantz., Euphorbiaceae), common bean (*Phaseolus vulgaris* L., Fabaceae), sesame (*Sesamum indicum* L., Pedaliaceae) and maize (*Zea mays* L., Poaceae) (Maes and Tellez Robleto 1988, Maes and Göllner-Scheiding 1993). These records pertain to incidental occurrences (resting specimens) and do not imply feeding associations (Carroll and Loye 1987, Aldrich et al. 1990b). The records of *Jadera haematoloma* feeding and even causing slight damage on bolls of cotton (*Gossypium hirsutum* L., Malvaceae) in Texas (Sanderson 1905, 1906) and Oklahoma (Drew and Schaefer 1963) are also doubtful.

Under laboratory conditions, *Jadera haematoloma* cultures can be maintained for several generations on seeds of *Koelreuteria paniculata* and water; seeds of *Cardiospermum corindum* and *Cardiospermum grandiflorum* were also successfully used for such purposes (Aldrich et al. 1990a, b). Sunflower seeds are suitable for rearing at least one generation (Aldrich et al. 1979).

Occasionally the bugs feed on various disabled or freshly dead arthropods (Carroll and Loye 1987). Cannibalism in the field or in captivity also is not rare. Entomophagy mostly involves larvae or reproductive females feeding on freshly moulted larvae or teneral adults, or much smaller larvae (Carroll and Loye 1987, Ribeiro 1989). First instar larvae often cannibalize eggs soon after hatching under laboratory conditions (Ribeiro 1989).

### Feeding

It feeds exclusively on the mature and nearly mature seeds of host plants (Carroll and Boyd 1992). Adult females feed more frequently than males (Carroll 1991). On host plants whose fruit is a bladdery capsule with seeds attached to the septum and situated close to the middle (*Koelreuteria*, *Cardiospermum*), adults, most frequently females, access the seeds through the pericarp using their rostrum; smaller males and larvae usually feed on fruits that are damaged or dehiscent and, therefore, open (Carroll and Boyd 1992). In Florida, oviposition sometimes occurs into the capsule of *Cardiospermum corindum* through small openings of the pericarp, and larvae develop there until at least the fourth instar (Carroll and Loye 1987, Carroll 1988).

The bugs cannot access seeds of *Sapindus oahuensis* through the fleshy, hardened drupe; therefore, it feeds only on the pericarp (Gagné 1971b). For similar reasons it does not colonize *Sapindus saponaria* in Florida; however, in captivity it readily feeds on manually opened seeds (Carroll and Loye 1987). In populations feeding on *Sapindus saponaria* var. *drummondii* in the southcentral part of the USA, young larvae cannot access to the seed; therefore, they are restricted to feeding on fallen and damaged fruits, but 4th–5th instar larvae and adults can penetrate the drupe with their labium (Carroll and Loye 1987).

### Coevolution with host plants

In several cases length of the labium differs significantly between populations on native host plants and nearby populations on introduced host plants. In some populations the change in the average length of the labium can be nearly 25%. The increase or decrease in the length of the labium is consistent with the difference in fruit size and morphology of the native and introduced hosts (Carroll and Boyd 1992). Cross-rearing experiments indicate that the differences are evolved rather than induced by developing on a particular host species (Carroll et al. 1997). Examination of museum specimens also provides evidence for such morphological changes and indicates directional selection and a rapid adaptive evolution in the relatively close past (20–50 years, about 100 generations) following the bug’s colonization of host plants introduced into its range. In some populations the allometric change is restricted to the labium; in other populations the shape of the head and the thorax is also slightly different. Laboratory crossing and backcrossing experiments indicate considerable additive genetic variation in length of the labium in populations on both native and introduced host plants; epistatic and dominance variance for the length of the labium was proven (Carroll and Boyd 1992, Carroll and Dingle 1996, Carroll et al. 2001, 2003a, Dingle et al. 2009). The length of the labium and the wing morph frequency also show genetic correlation (Dingle et al. 2009). The rate of microevolution in length of the labium was estimated by Hendry and Kinnison (1999) and Carroll et al. (2001).

### Aggregation

Feeding and reproductive adults and larvae form prominent, mixed-instar aggregations on host plants, most commonly on the trunks and on fallen seeds (Carroll and Loye 1987, Ribeiro 1989). The size of the aggregations can reach hundreds or thousands individuals on hosts with large seed crops (Carroll and Salamon 1995), but in warm temperate regions of southcentral USA (Oklahoma) they tend to be larger than those in tropical areas (southern Florida) (Carroll 1988). The tendency of larvae to disperse increases with age, but larvae of every instar rejoin aggregations to moult (Ribeiro 1989).

The structure of aggregations formed by diapausing adults in the canopies of goldenrain trees (*Koelreuteria* sp.) in Florida was studied by Zych (2010). Aggregations were composed of as many as 300 indivituals, usually on more terminal branches more or less parallel to the ground and always on the undersides of leaves.

### Phenology and lifespan

Data are available only from the USA (Carroll and Loye 1987, Carrol 1988). Populations in warm temperate regions of the southcentral part (Oklahoma) and tropical areas (southern Florida) differ greatly in phenology, partly due to thermal seasonality and partly to differences in host-plant phenology.

In Oklahoma (where the population feeds on *Koelreuteria paniculata* and *Sapindus saponaria* var. *drummondii* with seeds ripening in late July–August and mid-August–September, respectively) reproduction is highly seasonal. Adults and larvae overwinter in dense clusters, mostly on the ground among leaf litter. They leave their refugia around February or March, and overwintered females generally oviposit in March; then the overwintering adults decline in May and June. Adults of the new generation start to emerge in late July; mating and oviposition continue until early October. In October, while food is still available, they enter diapause (Carroll and Loye 1987, Carroll 1988), diapauses which cannot be interrupted (Carroll 1988).

In Florida (where the population feeds on *Cardiospermum corindum* with most seeds ripening in May and in November–December) it breeds year round. Adults start to feed and reproduce in late April and May, with bugs (mainly adults of the new generation) entering a starvation diapause in early summer when the seed base is exhausted. A second reproductive period follows from November until January. From January, as food again becomes unavailable, they enter starvation diapause, spending the period in clusters, mostly on herbaceous plants (Carroll and Loye 1987, Carrol 1988, Zych 2010). Diapausing individuals occasionally take nectar from flowering *Bidens* sp. (Asteraceae) or fluid from petioles of *Koelreuteria elegans* (Carroll and Loye 1987).

Individuals are inactive but the moulting of larvae is continuous during diapause in both populations (Carroll 1988). Reproductive adults may live for as long as 2 months (Carroll 1991).

### Development

Mean adult development time does not different between sexes (Carroll 1988). In Florida, development time on the native host plant (*Cardiospermum corindum*) is longer, age to first reproduction is longer than on the introduced host plant (*Koelreuteria elegans*) (Carroll 1988, Carroll et al. 1997, 2001). Survivorship of both populations is higher on the ‘home’ host plant, suggesting the existence of populations adapted to the introduced host (Carroll et al. 1998). Group effects were observed under laboratory conditions: young larvae reared in groups moulted significantly earlier and more synchronously than isolated larvae and mortality was lower (Ribeiro 1989).

### Population structure

Data are available only from the USA. Adult sex ratio in populations in humid parts of the southcentral region (Oklahoma) is generally strongly male-biased (ranging from 1:1 to 5:1, average 2.73±0.95 males per female), while in populations in tropical areas (southern Florida), it is close to 1:1 (Carroll 1988, 1993, Carroll and Corneli 1995, Carroll and Salamon 1995). The male-bias of the sex ratio in Oklahoma is mainly due to greater female mortality (Carroll 1988).

### Mating behaviour

Mating behaviour was studied in detail by Carroll (1988, 1991, 1993). Reproduction takes place within the aggregation. Males search for mates on the ground and in the canopy, but sit-and-wait searching also occurs, with males remaining stationary until they detect an approaching individual (Carroll 1991). After approaching, the male mounts the female’s back, attempts intromission, and, if successful, turns around and attains an end-to-end mating position. The pairs generally remain connected for several hours, but duration of copulation is highly variable (from 20 minutes up to 11 days with an average of 20.5±24.5 hours under laboratory conditions). The prolonged copulation is much longer than needed for sperm transfer alone and serves as postinsemination mate guarding (Carroll 1988, 1991). Female resistance appears not to have a major influence on the duration copulations (Carroll 1991, 1993, Carroll and Corneli 1995, Zych 2012).

Average duration of copulations of virgin females is significantly shorter than those of the same females during subsequent copulations. Under laboratory conditions, duration of the copulation tends to be greater in groups where sex ratios are more male biased because of intense male–male competition (Carroll 1991). In male-biased populations there is strong positive sexual selection for male body size. In such populations the ratio of large to small males mating mating is greater. Similarly, mating males are significantly larger than single males. These differences are not observed in unbiased populations. The mating advantage of large males results from their increased locomotion activity (Carroll and Salamon 1995)

### Oviposition

Eggs generally are laid in a hole about 1 cm deep, which the female digs with its fore legs in dry soil close to the host tree. After completing oviposition, the female covers the eggs with soil using its fore legs (Sanderson 1906, Carroll 1988, 1991). In Florida, oviposition also commonly occurs into the capsule of *Cardiospermum corindum* through emergence holes made by lepidopteran larvae (Carroll 1988); similar behaviour was observed in Texas (Sanderson 1906). The male interrupts copulation but climbs the back of the female and guards it during oviposition, holding its phallus close to the female’s vulva. Ovipositing females are commonly targets of searching males, but the guarding male usually effectively prevents takeover by quickly recopulating (Carroll 1991).

Egg clutches typically are laid at 1- to 2-day intervals for 2–3 weeks; a clutch contains 1–20 eggs (14±4.1 in average) in Oklahoma (Carroll 1988, 1991, 1993). Maximum lifetime fecundity is estimated to be 400–800 eggs. After oviposition, pairs usually recouple, but males generally guard mates for only one or two ovipositions. In male-biased populations males guard their mates significantly more frequently than those from unbiased populations (Carroll 1993, Carroll and Corneli 1995).

If the male departs it remains sexually active, and often mounts the next available female encountered. Most females also copulate with several males (Carroll 1991, 1993). Maximum lifespan of twice-mated females after the last egg is laid is about 6 days (Carroll 1991).

In Oklahoma, females produce significantly more and smaller eggs than those from southern Florida (Carroll et al. 1998). Florida females on the native host plant (*Cardiospermum corindum*) produce significantly larger eggs than those on the introduced host plant (*Koelreuteria elegans*). However, egg production of females from populations on the native host is the same on native or introduced hosts, whereas females from populations on the introduced host lay significantly fewer eggs per day on the native host but exhibit enhanced fecundity on the introduced host. This suggests the existence of populations adapted to the introduced host (Carroll et al. 1998).

### Aposematism, natural enemies and interspecific competitors

The conspicuous aggregations of the red larvae are aposematic. Laboratory experiments with toads (various *Bufo* spp., Bufonidae) and blue jays (*Cyanocitta cristata* (Linnaeus, 1758), Corvidae), as well as field observations on Mantidae, showed that after having tasted larvae these predators avoided other larvae. Although adults also are distasteful, their effectiveness alone in causing avoidance is uncertain (Ribeiro 1989, Aldrich et al. 1990a).

In U.S. populations there is little or no predation on the bugs (Aldrich et al. 1990a) and no parasitoids have been observed at any phase of the life cycle (Carroll 1988). Caterpillars of two lycaenid butterflies, *Chlorostrymon maesites* (Herrich-Schäffer, 1865) and *Cyclargus thomasi* (Clench, 1941), consume immature seeds of *Cardiospermum corindum* in southern Florida, and because they cause considerable (occasionally more than 50%) loss in production, they are likely to be significant interspecific competitors of *Jadera haematoloma* (Carroll 1988).

### Allomones, sequestration, attractants

Pinching the bugs causes them to discharge haemolymph from the rostrum and intersegmentally, and also to emit secretions from the scent glands (Aldrich et al. 1990a). Dorsal abdominal scent glands persist and they are functional in the adult. The volatile compounds of scent gland secretions were analyzed by Aldrich et al. (1979, 1990b). In addition to (*E*) -2-hexenal and (*E*) -2-octenal, compounds often found in Heteroptera, several monoterpene hydrocarbons were identified. The secretion is not sexually different, but compounds from glands of segment IV and V differ: unsaturated carbonyl compounds are produced only by the anterior and monoterpene hydrocarbons only by the posterior gland. Secretions from the ventral abdominal gland of the male again differ from those of the dorsal abdominal glands (Aldrich et al. 1990b). No clear alarm-releasing activity of the compounds on larvae could be proven (Aldrich et al. 1990b).

The haemolymph of *Jadera haematoloma* sequesters glycosides. These are not truly cyanogenic; HCN is released from crushed individuals only if they were reared on *Cardiospermum grandiflorum* and if *β*-glucosidase is added (Aldrich et al. 1990a). Feces of individuals that develop on *Koelreuteria paniculata* contains 4-methyl-2 (5H) -furanone, which attracts conspecific individuals (Aldrich 1990a).

### Stridulation

Stridulation was recorded and documented by Zych et al. (2012). A raised surface at the lateral margin of abdominal tergite I functions as a plectrum; fused abdominal tergites I+II are moved rapidly (15–25 Hz) anteriorly and posteriorly, opposing partly the posterior edge of metanotum, partly the ventral side of the anterior margin of the wing functioning as stridulitrum. Thus a low-frequency and a high-frequency signal, respectively, are produced. Sound producing structures are present and sound is produced in both sexes. The sound is produced as a response to a rapidly approaching conspecific individual, especially if it climbs on the top of the signaller. Apparently sound indicates that the female or male is unreceptive for mating. Interspecific encounters or other threat stimuli do not elicit signals (Zych et al. 2012).

### Pest status, control

Large populations around habitations may alarm people (Mead 1985); it was documented in Texas and Oklahoma as a nuisance insect, occasionally entering houses especially in the summer and early autumn (Wheeler 1982 cited by Mead 1985, Reinert et al. 1999). No control measures are necessary. Removing the fallen seeds from under trees and manual collecting and destroying the bugs are usually enough in case they are a nuisance. If chemical control is needed, diazinon EC could be effective (Mead 1985). Biocontrol products containing formulations of *Beauveria bassana* Vuill. strains show promise for a low-impact and environmentally sound control (Reinert et al. 1999).

### Distribution

**Figure 6.**
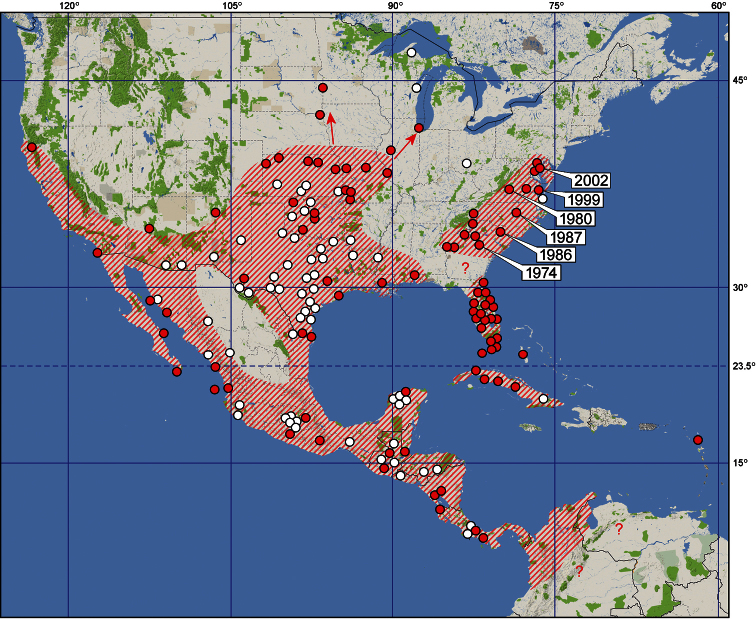
Distribution of *Jadera haematoloma* in the Americas. Red dots represent literature records, white dots represent localities mapped by Carroll (1988) without available locality names. Dashed line indicates uncertainty in the border of the area. Numbers along the Atlantic Coast of the USA indicate the years of first captures from the indicated localities.

The distribution range of *Jadera haematoloma* is determined by the geography of its native and introduced host plant species (Carroll 1988). It is the only species of the genus that enters temperate regions of North America (Fig. 6). It occurs throughout the Gulf Plain, and northward it broadly extends into the area of mixed open forests and temperate grasslands in the western part of the Interior Plains. The northern extent of its range is somewhat indistinct because only a few scattered records are available from the Great Lakes region; these records most likely represent isolated adventitious individuals rather than established populations. The bug does not enter the regions characterized by semi-desert and shrubland vegetation in the Western Mountains and Mexican Plateau, but its range is more or less continuous throughout the subtropical and tropical forests of southern Mexico and Central America.

Although *Jadera haematoloma* is common in the peninsular part of Florida, it does not enter the ‘panhandle’; therefore, this population is apparently disjunct from that of the southcentral USA (cf. Carroll 1988). No published records are known from the Atlantic Plain prior to 1974, although museum specimens indicate its presence in Virginia as early as 1932 (S.P. Carroll, *pers. comm*.). In the 1970s it apparently started to gradually expand along the Atlantic Coast towards the northeast (Hoffman and Steiner 2005); the northernmost published localities are in Maryland. It does not occur in higher parts of the Appalachian Highlands.

Records from northern South America are scarce, but most likely its area is bordered in the south by the Northern Andes.

Records from the sub-Amazonian South America, e.g. southern Brazil (Banho et al. 2011), Uruguay, Paraguay (Berg 1892), Argentina (Pennington 1922, Carroll and Dingle 1996, Bressa et al. 2001, Pall and Coscarón 2012) are apparently erroneous and probably at least partly pertain to the superficially similar *Jadera pyrrholoma* Stål, 1870 (Göllner-Scheiding 1979). The recent record from Buenos Aires is accompanied with a photo (Pall and Coscarón 2012: 1447, fig. 4F). The much broader pronotum and fore wing, the rather distinct dark dots on the pronotum and the different shape of the marginal vitta, and the reddish head of the specimen are sufficient to exclude the possibility that the photographed specimen is *Jadera haematoloma*. It apparently represents *Jadera pectoralis* Stål, 1862 or *Jadera parapectoralis* Göllner-Scheiding, 1979 (opinion confirmed by U. Göllner-Scheiding *in litt*.).

*Jadera haematoloma* colonizes several islands of the Caribbean. The single record from Antigua is based on an incompletely coloured specimen, the record therefore is uncertain (Barber 1923), but its occurrence on Antigua is likely. It was inadvertently introduced to Hawaii in the 1960s; it was detected on O’ahu Island in August 1968 (Davis 1969) and subsequently recorded on Kauai and Kona Islands (Gagné 1971a, b). It is recorded for the first time from Southeast Asia (Taiwan) in the present paper.

Because several sapindaceans are cultivated widely as ornamental trees, eventual introduction of *Jadera haematoloma* likely will result in the colonization of new areas.

**USA. California**: Coronado Is. (Van Duzee 1923); unspecified locality (Uhler 1872, 1876, Göllner-Scheiding 1979); **Arizona**: Yavapai Co.: Prescott (Carroll and Boyd 1992); unspecified locality (Uhler 1872, 1876, Aldrich et al. 1990b); **New Mexico** (Carroll 1988, in map); **Colorado** (Uhler 1872, 1876, Gillette and Baker 1895); **Kansas**: Douglas, Riley, Cloud, Decatur and Sherman Counties (Leay 1928); unspecified localities (Göllner-Scheiding 1979); **Oklahoma**: Cleveland, Cotton, Oklahoma and Woodward Counties (Carroll 1988, Carroll and Boyd 1992); unspecified locality (Drew and Schaefer 1963, Göllner-Scheiding 1979); **Texas**: Brownsville (Barber 1906, Malloch 1918, Torre-Bueno 1930); Navasota (Sanderson 1906); Galveston (Snow 1906); Fort Davis (Torre-Bueno 1931); Hidalgo Co.: Bentsen-Rio Grande Valley State Park (Carroll and Boyd 1992); unspecified locality (Stål 1870, Porter 1917, Göllner-Scheiding 1979); **Arkansas** (Carroll 1988, in map); **Missouri**: Barry, Boone, Jackson, Jasper, Lawrence and St. Louis Counties; unspecified locality (Göllner-Scheiding 1979); **Louisiana**: Baton Rouge (Carroll and Boyd 1992); unspecified locality (Göllner-Scheiding 1979); **Tennessee** (Hoffman and Steiner 2005); **Alabama**: Mobile (Blatchley 1926); **Florida**: Sanford (Van Duzee 1909); Lakeland, Everglade, Chokoloskee (Barber 1914); Leesburg (McLennan et al. 1994, Carroll et al. 1997, 1998, 2003b, Winchell et al. 2000); Fort Myers (Carroll et al. 1997, 1998); Gainesville (Carroll et al. 2003b, Zych 2010, Zych et al. 2012); Lake Wales (Carroll and Boyd 1992, Carroll et al. 1997, 1998, 2003b); Duval, St. Johns, Flagler, Marion, Volusia, Lake, Seminole, Orange, Brevard, Hernando, Pasco, Pinellas, Hillsborough, Polk, Manatee, Sarasota, Hardee, Highlands, Okeechobee, St. Lucia, Lee, Monroe and Miami-Dade Counties (Mead 1985, in map); Florida Keys (Barber 1914, Aldrich et al. 1979, Carroll 1988, Carroll and Boyd 1992, Winchell et al. 2000, Carroll et al. 1997, 2003b); **Georgia**: Clarke, Lamar, Richmond and Troup Counties (Hoffman and Steiner 2005); **South Carolina**: Darlington, McCormick, and Pickens Counties (Hoffman and Steiner 2005); **North Carolina**: Biltmore (Brimley 1938); Wake County: Raleigh (Hoffman and Steiner 2005); unspecified locality (Sherman 1948); **Virginia**: Bedford County: Boonsboro; Alexandria; Mathews and Henrico Counties (Hoffman, Melber 2005); Cape Henry (S.P. Carroll, *pers. comm*.); unspecified locality (Carroll 1988, in map); **Maryland**: Howard County: Marriottsville; Queen Annes County: Parole (Hoffman, Melber 2005); **Illinois**: Havana (Malloch 1918); Chicago (introduced) (Blatchley 1926); **Iowa** (Slater and Baranowski 1978); **South Dakota**: Elk Point; Lake Hendricks (Harris 1937); **Ohio** (Carroll 1988, map); **Wisconsin** (Carroll 1988, map); **Michigan** (Carroll 1988, in map); **Hawaii** (introduced): O’ahu: Waipahu (Davis 1969); Kauai; Kona (Gagné 1971a, b). — **THE BAHAMAS** (Carroll and Boyd 1992, Carroll and Loye 2012). — **CUBA.** Havana (Guérin-Méneville 1857, Alayo 1967, Grillo and Alayo 1978); Ariguanabo (Alayo 1967); Camagüey Prov.: Baraguá (Barber and Bruner 1947); Las Villas [= Cienfuegos Prov., part]: Cienfuegos: Soledad (Grillo and Alayo 1978); Matanzas Prov.: Cienaga de Zapata, Central Mercedes (Alayo 1967, Grillo and Alayo 1978); Isle of Pines [= Isla de la Juventud]; unspecified locality (Stål 1870, Carroll and Boyd 1992). — **ANTIGUA AND BARBUDA.** Antigua (Barber 1923). — **MEXICO.** Oajaca [= Oaxaca] (Walker 1872, as *Lygaeus marginalis*); Islas Marías; Jalisco: San Blas; Chihuahua: Pinos Altos; Guerrero: Chilpancingo, Omilteme [= Omiltemi], Xucumanatlan; Yucatán: Temax (Distant 1893); Sinaloa: Mazatlán (Distant 1893, H. Brailovsky *pers. comm*.); Cape St. Lucas (Uhler 1894); Guaymas; Carmen Is.; Tiburón Is. (Van Duzee 1923); Puebla (McLennan et al. 1994, Carroll and Loye 2012); Yucatán Peninsula (Carroll and Boyd 1992); Baja California (Carroll and Loye 2012); Jalisco: Guadalajara, Puerto Vallarta; Morelos: Tepoztlán, Cuautla; Oaxaca: Huajuapan de León, Montealbán; Estado de México: Chalma; Puebla: Acatlán; Guerrero: 10 km Carr. Cacahuamilpa-Taxco (Acuitlapan), Acahuizotla, Granados; Michoacan: Tingambato; Nuevo León: Monterrey, Ladera Oeste del Cerro de la Silla; Nayarit: Compostela (H. Brailovsky, *pers. comm*.); unspecified locality (Herrich-Schäffer 1847, Blöte 1934, Göllner-Scheiding 1979, 1983). All records before 1982 are doubtful because of possible confusion with *Jadera diaphona*. — **GUATEMALA.** San Gerónimo, Dueñas (Distant 1882); Capetillo: El Reposo (Distant 1893). — **BELIZE.** “British Honduras” [= Belize]: Sarstoon River (Distant 1893). — **EL SALVADOR** (Carroll 1988, in map). — **HONDURAS** (Carroll 1988, in map). — **NICARAGUA.** Managua; Boaco (Maes and Göllner-Scheiding 1993). — **COSTA RICA.** Guanancaste: Santa Rosa National Park, Playa Naranjo (Arnold 1995). — **PANAMA.** Chiriquí: Tolé; Volcán de Chiriqui [= Volcán Barú] (Distant 1893). — **COLUMBIA** (Dallas 1852, Göllner-Scheiding 1979, 1983, Henry and Froeschner 1988). — **VENEZUELA** (Göllner-Scheiding 1979, 1983, Henry and Froeschner 1988). — **TAIWAN. Chiayi County**: Zhuchi Township, Shihjhuo (present study); **Tainan City**: East District (present study); **Kaohsiung City**: Ciaotou; Cishan; Dagangshan Scenic Area; Nanzih (present study).

## Material and methods

Populations of *Jadera haematoloma* were observed at 7 sites in Kaohsiung City, southern Taiwan (J.F. Tsai, Y.X. Hsieh, November 2012–January 2013) and at one site in Tainan City (January 2013, U. Ong). Single individuals were recorded from two additional localities.

Specimens were examined using a SteREO Discovery.V20 microscope with a PlanApo S 1.0x FWD 60mm objective. Measurements of larvae were taken using a calibrated Leica stage micrometer (10310345); they were preserved by freezing in order to maintain their shape. Photographs were taken with Nikon D300 and Canon EOS 5D digital cameras equipped with AF-S Nikkor 60mm micro-lens and MPE-65 mm lens, respectively.

Measurements of populations of specimens were compared using non-parametric Wilcoxon–Mann–Whitney two-sample rank-sum test; all presented *U* and *p* values were obtained using this test.

Plant names are used following the online database of the International Plant Names Index (www.ipni.org, accessed December 2012).

Voucher specimens of *Jadera haematoloma* collected during the present study have been deposited in the following public collections: National Museum of Natural Science, Taichung, Taiwan; Taiwan Forestry Research Institution, Taipei, Taiwan; Department of Entomology, National Taiwan University, Taipei, Taiwan; Department of Entomology, National Chung Hsing University, Taichung, Taiwan; Taiwan Agricultural Research Institute, Taichung, Taiwan; Department of Plant Medicine, National Pingtung University of Science and Technology, Neipu, Taiwan; Department of Entomology, Nankai University, Tianjin, China; Hungarian Natural History Museum, Budapest, Hungary; Department of Entomology, National Museum, Prague, Czech Republic.

## Results

### Distribution and habitat in Taiwan

Single individuals of *Jadera haematoloma* were observed at the following localities:

Kaohsiung City: Alian District, Dagangshan Scenic Area, at Huashi Pavilion, on the way to Chaofeng Temple, 30.viii.2012, Y.X. Hsieh, J.X. Fang. A secondary forest with *Bauhinia variegata* L. (Fabaceae) as dominant, mixed with several cultivated plants, most importantly *Broussonetia papyrifera* (L.) Vent. (Moraceae), *Mallotus japonicus* Müll.Arg. and *Bischofia javanica* Blume (both Euphorbiaceae). The specimen was observed on *Miscanthus* sp. (Poaceae).

Chiayi County: Zhuchi Township, Shihjhuo (23°28'27"N, 120°42'03"E), 1300 m a.s.l., 5.xi.2012, S.F. Yang. Digital photo of a single specimen was taken and provided to us by S.F. Yang.

Populations of *Jadera haematoloma* were collected or observed at the following sites (Table 2):

**Site 1.** Kaohsiung City: Cishan, Ci-nan Third Road (22°49'23"N, 120°27'44.17"E), 30.xi.2012, Y.X. Hsieh, J.F. Tsai (Fig. 21). Around a lychee (*Litchi chinensis* Sonn., Sapindaceae) orchard. The orchard was bordered by a chain-link fence climbed by several plants, the dominant among them was the heartseed vine (*Cardiospermum halicacabum*), mixed with some *Passiflora foetida* L. (Passifloraceae), *Mikania micrantha* Kunth and *Bidens pilosa* L. var.*radiata* Sch.Bip. (both Asteraceae).The fallen leaves of the litchee trees were removed from under the trees and moved to the margin of the orchard under the fence. Several macropterous and brachypterous adults and larvae of all instars were observed to actively walk on and in the leaf litter and feed on *Cardiospermum halicacabum*.

**Site 2.** Kaohsiung City: Cishan, a residential area along Ci-ping First Road (22°52'56"N, 120°29'46"E), 2.xii.2012, Y.X. Hsieh. An empty yard with two patches of *Cardiospermum halicacabum*. Adults with first and fourth instar larvae were observed.

**Site 3.** Kaohsiung City: Ciaotou, corner of Gong-yuan Road and Ciao-chung Street (22°45'23"N, 120°18'30"E), 30.xi.2012, Y.X. Hsieh, J.F. Tsai. A vegetable garden bordered by a plastic mesh fence fixed to cemented pillars, climbed by *Cardiospermum halicacabum* only, with a layer of dead cucurbitacean leaves under the fence (the garden was apparently used for growing melon earlier). Adults and larvae (first to third instars) were observed mainly on the heartseed vine, only a few specimens in the leaf litter under the plant. Several mating pairs and brachypterous individuals were found.

**Site 4.** Kaoshiung City: Ciaotou, at the junction of Shu-he Road and Tong-shu Road (22°45'17"N, 120°18'16"E), 30.xi.2012, Y.X. Hsieh, J.F. Tsai. A fallow ground owned by Taiwan Sugar Corporation, with some herbs on the ground, among them *Cardiospermum halicacabum*. Several adults, including mating pairs were observed on 16.xi.2012 by Y.X. Hsieh, but the abundance of adults was very low two weeks later: only 7 adults were collected; however, about a hundred larvae were found.

**Site 5.** Kaohsiung City: Ciaotou (22°44'27"N, 120°19'24"E), 30.xi.2012, Y.X. Hsieh, J.F. Tsai. A flower farm of Taiwan Sugar Corporation; a public recreation farm with several cultivated vegetables, flowers and trees. Several adults and 2nd–4th instar larvae were collected in an old-growth patch of *Koelreuteria elegans* subsp. *formosana* with a thick layer of fallen leaves and seed pods under the trees. Several dozens of adults were collected by Y.H. Hsieh at the same locality on 3.xii.2012. One month later (14.i.2013, Y.X. Hsieh) hundreds of adults (clearly more males than females), including several mating pairs, and larvae of all instars forming aggregations near the base of the trunks were observed. Careful searching on all dates yielded no brachypterous individuals.

**Site 6.** Kaoshiung City: Nanzih (22°43'51"N, 120°20'08"E), 3.xii.2012, Y.X. Hsieh. A patch of *Koelreuteria elegans* subsp. *formosana* trees (with mature fruits in this season) planted along the street, opposite the building of Kaohsiung High Administrative Court. Adults were actively walking and feeding on the seeds on and among the fallen leaves and fruits under the tree.

**Site 7.** Kaohsiung City: Nanzih. Kaohsiung Metropolitan Park (22°43'57.08"N, 120°19'0.71"E), 10.xii.2012, Y.X. Hsieh. A patch of *Koelreuteria elegans* subsp. *formosana* trees (with mature fruits) close to the baseball field. Several mating pairs were observed on the trunk of the trees near the ground; all females were gravid. A careful search yielded no larvae or brachypterous adults.

**Site 8.** Tainan City: East District, near Sheng-chen Road (22°57'55.28"N, 120°13'23.33"E), 13.i.2013, U. Ong. A large fallow ground owned by Taiwan Sugar Corporation, with a large number of *Cardiospermum halicacabum* mixed with *Bidens pilosa* var. *radiata*. A large number of adults, including several mating pairs, and larvae were observed feeding on *Cardiospermum halicacabum* and nectar of *Bidens pilosa*. Brachypters were much more abundant than macropters.

**Table 2.** Collected individuals of *Jadera haematoloma* in the investigated sites of Kaohsiung City and Tainan City (for description of the sites see text).<br/>

	**male**		**female**		**host**
	**brachypterous**	**macropterous**	**brachypterous**	**macropterous**	
**Site 1**					*Cardiospermum halicacabum*
21.xi.2012		3		6	
22.xi.2012		3		5	
26.xi.2012		1	1	2	
30.xi.2012	3	25	2	14	
**Site 3**					*Cardiospermum halicacabum*
22.xi.2012	4	3	1	2	
30.xi.2012	2	7	2	5	
**Site 4**					*Cardiospermum halicacabum*
16.xi.2012		4		6	
30.xi.2012	3	1	1	2	
**Site 5**					*Koelreuteria elegans* subsp.*formosana*
30.xi.2012		9		2	
3.xii.2012		39		17	
14.i.2013		42		14	
**Site 6**					*Koelreuteria elegans* subsp.*formosana*
3.xii.2012		3		1	
**Site 7**					*Koelreuteria elegans* subsp.*formosana*
10.xii.2012		6		6	
**Site 8**					*Cardiospermum halicacabum*
13. i.2013	20	5	12	3	

## Morphology

### Intraspecific variation of adult

**Colour.** Only slight variation in the colour was observed. In males middle portion of abdominal sternites II–VI was usually black, but several specimens, especially females, had sternites III–VI more or less broadly margined with red posteriorly (Fig. 2) as reported by Göllner-Scheiding (1979).

**Body measurements.** Adults (*n* = 187) from various localities in Kaohsiung were measured (Table 3). Body length of males was significantly smaller than females in both the macropterous (*U* = 16.74, *p* < 0.001) and brachypterous (*U* = 6.45, *p* < 0.001) specimens. Width of pronotum of males was also significantly smaller than that of females in both the macropterous (*U* = 16.93, *p* < 0.001) and brachypterous (*U* = 6.52, *p* < 0.001) individuals. Humeral width of pronotum of macropterous specimens was significantly larger than that of brachypterous individuals in both males (*U* = 5.26, *p* < 0.001) and females (*U* = 4.30, *p* < 0.001).

**Table 3.** Measurements (in mm) and relative length of the labium of macropterous and brachypterous adults.<br/>

	**body length**		**width of pronotum**		**relative length of labium**^1^
	**range**	**average, SD**	**range**	**average, SD**	
**males**					
macropterous (*N* = 101)	9.37–12.01	10.60±0.56	2.64–3.30	2.98±0.16	II-P to IV-P^2^
brachypterous (*N* = 8)	8.58–9.10	8.71±0.29	2.51–2.90	2.74±0.14	III-A to IV-A^3^
**females**					
macropterous (*N* = 68)	10.16–12.80	11.67±0.66	2.90–3.96	3.30±0.20	III-M to IV-P^4^
brachypterous (*N* = 7)	9.24–10.82	9.67±0.60	2.90–3.56	3.19±0.23	III-P

^1^ The position of the apex of the labium in respect to the abdominal sternites is given; Roman numerals refer to the segmental homology; A = anterior half; P = posterior half.

^2^ II-P: 5, III-A: 43, III-P: 38, IV-A: 8, IV-P: 1. Five specimens excluded.

^3^ III-A: 6, III-P: 1, IV-A: 1.

^4^ III-M: 26, III-P: 15, IV-A: 16, IV- P: 3. Eight specimens excluded.

Adults collected on two different host plants (*Cardiospermum halicacabum*, *Koelreuteria elegans* subsp. *formosana*) at various sites in Kaohsiung were compared (Table 4). Males collected on *Cardiospermum halicacabum* were slightly smaller on average than those collected on *Koelreuteria elegans* subsp. *formosana*, but neither the difference in total length (*U* = 0.69, *p* = 0.488), nor length measured from apex of the clypeus to the apex of abdomen (*U* = 0.93, *p* = 0.353) was statistically significant. Females collected on *Cardiospermum halicacabum* were slightly larger on average than those collected on *Koelreuteria elegans* subsp. *formosana* (*U* = 0.93, *p* = 0.353) in respect to total length, but the relationship was opposite (*U* = 1.89, *p* = 0.059) when measuring from apex of the clypeus to the apex of abdomen; these differences are also not statistically significant. The fact that on one of the host plants the mean total lengths of specimens of one sex were greater than those of the opposite sex also suggests that there is no substantial difference in the body size of individuals from the two host plants. Measurements to apex of abdomen might reveal comparative differences in food level, hydration or reproductive condition of females. However, because it is very plastic, it is not as useful a measure for assessing developmental or genetic size differences among adults within or between populations.

**Variation in the relative length of labium.** The same specimens as in the previous paragraph were examined (Table 3). The apex of the labium in resting position attains at least the posterior margin of sternite II (♂) or the middle of abdominal sternite III (♀), and in extreme cases it approaches the posterior margin of abdominal sternite IV (♂, ♀). Both macropterous and brachypterous females had a relatively longer labium on average than the males. In both sexes macropterous individuals had a relatively longer labium on average than brachypterous individuals of the same sex. The relative length of the labium seems to be slightly longer in both males and females of the populations on *Cardiospermum halicacabum* than those on *Koelreuteria elegans* subsp. *formosana* (Table 4), but no conclusion can be drawn for our data and careful testing is needed based on absolute lengths.

**Table 4.** Body size (in mm) and relative length of the labium in specimens of different sex collected from different host plants (all macropterous).<br/>

	***Cardiospermum halicacabum***			***Koelreuteria elegans* subsp.*formosana***		
	**body length (head–wing)**	**body length (head–abdomen)**	**labium**	**body length (head–wing)**	**body length (head–abdomen)**	**labium**^1^
**males**	10.56±0.58<br/> (9.50–12.01) <br/> *N* = 47	8.93±0.34<br/> (8.32–9.50) <br/> *N* = 49	II-P (*N* = 2) <br/> III-A (*N* = 14) <br/> III-P (*N* = 17) <br/> IV-A (*N* = 7) <br/> IV-P (*N* = 1)	10.64±0.55<br/> (9.37–11.88) <br/> *N* = 54	9.02±0.40<br/> (8.05–9.90) <br/> *N* = 49	II-P (*N* = 3) <br/> III-A (*N* = 29) <br/> III-P (*N* = 21) <br/> IV-A (*N* = 1)
**females**	11.72±0.70<br/> (10.16–12.80) <br/> *N* = 43	9.88±0.63<br/> (8.84–11.22) <br/> *N* = 39	III-A (*N* = 1) <br/> III-P (*N* = 18) <br/> IV-A (*N* = 14) <br/> IV-P (*N* = 1)	11.59±0.59<br/> (10.16–12.41) <br/> *N* = 25	10.19±0.53<br/> (9.37–10.96) <br/> *N* = 19	III-A (*N* = 12) <br/> III-P (*N* = 10) <br/> IV-A (*N* = 2) <br/> IV-P (*N* = 1)

^1^ The position of the apex of the labium in respect to the abdominal segments (sternites) is given; Roman numerals refer to the segmental homology; A = anterior half; P = posterior half.

**Wing polymorphism.** At most sites macropterous (Figs 1–2) and brachypterous (3–5) specimens were observed and collected too. Forty-four adults were counted at site 1 on 30.xi.2012; 5 (11.4%) were brachypterous. On one occasion (site 8, 13.i.2013) the vast majority (32 of 40, 80%) of the observed specimens were brachypterous (Table 2). No conspicuous difference was observed in the frequency of brachypterous individuals between the two sexes. Brachypterous specimens were observed only on *Cardiospermum halicacabum* and never on *Koelreuteria elegans* subsp. *formosana* (Table 2).

Slight variability was observed in the development of the fore wing of the brachypterous individuals. The apex of the wing can reach the anterior (Fig. 5) or posterior portion (Figs 3–4) of abdominal sternite VI; in some individuals the membrane is rather broad and subtriangular (Fig. 3), shorter and broadly rounded in others (Fig. 4), and in others is reduced to a narrow band (Fig. 5).

### Morphology of larvae

#### Diagnosis

Larvae of Rhopalidae can readily be recognized using the family keys of Jordan (1951), Leston and Scudder (1956), Herring and Ashlock (1971), or Yonke (1991); their unique diagnostic character is the posterior margin of abdominal tergite V deeply emarginate cranially; therefore, the abdominal tergite is longitudinally shortened along midline. Larvae of *Jadera haematoloma* are more or less similar in size, colour, and shape to those of two *Leptocoris* species, *Leptocoris augur* (Fabricius, 1781) and *Leptocoris vicinus* (Dallas, 1852), both native and common in Taiwan. The diagnostic characters of the three species are provided in Table 5.

#### Description

**Colour.** Body bright red (1st instar, Figs 7–8), or bright red with prothorax, pterothoracic tergum and pleuron reddish gray, pterothoracic sternum reddish (2nd–5th instars, Figs 9–20); antenna and legs pale (1st instar) to dark gray (2nd–5th instars), with more or less reddish shade, especially in younger instars, extremities of antennal segments usually more distinctly red at intersegmental articulations.

**Integument and vestiture.** Smooth, subshining, weakly sclerotized (1st instar) or dull, head, prothorax, pterothoracic tergum and pleuron more strongly sclerotized than abdomen, dorsal surface of head and thorax pruinose, ventral surface of head and prothorax together with thoracic pleuron and all coxae more strongly pruinose (2nd–5th instars); body sparsely covered with strong, stiff, almost bristle-like, pale (1st instar) or black (2nd–5th instar) setae.

**Head and cephalic appendages.** *Head* pentagonal; dorsal surface provided with several setae, with a series of discontinuously arranged setae along dorsal margin of eye, ventral surface without setae; vertex rounded and convex; V-shaped ecdysial suture distinct;clypeus simple, elevated above level of mandibular plates, pilose, with a tuft of setae at apex, apically broadened;mandibular plate thick, with a row of seate dorsally, laterally straight, not reaching apex of clypeus; antennifer situated in front of eye, visible in dorsal view, antenniferous tubercle distinct, with a tuft of setae;buccula undeveloped (1st–5th instars); eye rounded, prominent, distinctly separated from pronotum by a relatively long postocular margin provided with a single series of setae.*Antenna* with segments I–III subcylindrical, segment IV distinctly spindle-shaped in younger (1st–2nd) instars, gradually becoming subcylindrical in older (3rd–5th) instars.*Labium* and its individual segments of variable length; segment I slightly shorter than remaining segments, reaching or slightly surpassing posterior margin of eye but never reaching base of head (1st–5th instars); segment IV distinctly longer than remaining segments (1st–5th instars); labium of newly hatched larvae reaching apex of abdomen (Fig. 8), relative length of labium gradually becoming shorter from 1st to 5th instar, but individual variability great (Table 7).

**Thorax and thoracic appendages.***Prothorax*: pronotum broader than long, more or less trapeziform, with distinct anterior collar (1st–5th instars), humeri rounded, not protruded; prothoracic acetabula open posteriorly; *mesonotum* rectangular (1st–2nd instars), slightly expanded laterally (3rd instar), with well-developed mesothoracic wing pads reaching posterior margin of abdominal tergite I to middle of tergite II (4th instar) or posterior margin of tergite II to posterior half of tergite IV (5th instar); scutellar pad distinct in 4th and 5th instars; *mesosternum* flat; *metanotum* simple (1st–3rd instars) or with well developed metathoracic wing pads (4th–5th instars); *metasternum* large, subhexagonal, plate-like. *Legs* simple, setose, distance between fore and mid legs greater than that of mid and hind legs; distance between fore coxae much smaller that of mid coxae, distance between hind coxae somewhat greater than that of mid coxae.

**Abdomen** composed of 11 visible segments (tergites I and II distinct, sternite I absent); venter distinctly more convex than dorsum. *Dorsal abdominal scent glands* with two single minute openings situated between tergites IV/V and V/VI, intersegmental suture between tergites IV and V nearly straight, that between tergites V and VI deeply curved anteriad along midline; therefore, tergite V short along midline and gland openings situated close to each other; spiracles II–VIII situated posterolaterally on the respective sternites; trichobothrial formula 0-0-0-3-3-2 (sternites II–VII) in all stages; trichobothria on sternites III and IV situated submedially (rarely 3+4 trichobothria present on sternite IV), trichobothria of sternites V–VII situated on anterior portion of respective sternites, arranged transversely; genital segment distinguishable in 5th instars of both sexes: posterior margin of sternite VIII with slight (4th instar) to deep (5th instar) incision along midline, sternite IX depressed in female, abdominal sternite IX undivided (4th–5th instars), much swollen (5th instar) in male; ring-like segment XI usually exposed.

**Table 5.** Diagnostic characters for older larvae (3rd–5th instars) of *Jadera haematoloma* and two sympatric serinethines, *Leptocoris augur* and *Leptocoris vicinus*.<br/>

	***Jadera haematoloma* (Herrich-Schäffer, 1847)**	***Leptocoris augur* (Fabricius, 1781)**	***Leptocoris vicinus* (Dallas, 1852)**
1	Body bright red, head and thorax darker and conspicuously pruinose especially ventrally (Figs 7–20).	Body bright orange (Fig. 31: arrow, 34: arrow), head and thorax somewhat darker, body with weak or indistinct pruinosity.	Body colour similar to *Jadera haematoloma* but frequently darker red, body without pruniosity.
2	Mandibular plates broadly rounded distally, portion of head anteriad of antenniferous tubercles broadly truncate anteriorly.		Mandibular plates strongly narrowed distally, portion of head anteriad of antenniferous tubercles broadly rounded anteriorly.
3	With a single, broadly interrupted series of setae along dorsal margin of eye.	With a single, uninterrupted series of setae along dorsal margin of eye.	
4	Ecdysial suture of head V-shaped.	Ecdysial suture of head rather U-shaped, with its contralateral branches less diverging.	
5	Postocular portion of head of somewhat angulate lateral outline in dorsal view, provided with a single series of setae at each side, without protuberance.	Postocular portion of head of rounded lateral outline in dorsal view, provided with at least two irregular series of setae or irregular pilosity at each side, with a pair of blunt, angular protuberance dorsolaterally.	
6	Apex of labial segment I reaching posterior margin of eye.	Apex of labial segment I reaching base of head.	Apex of labial segment I extending to postocular part of head, approaching base of head.
7	All legs uniformly grey to black.	Coxae red to brownish, remaining segments of legs chestnut-coloured to black.	Coxae brownish red, remaining segments of legs black.
8	Intersegmental suture IV/V almost straight.	Intersegmental suture IV/V slightly curved posteriad at middle.	Intersegmental suture IV/V strongly curved posteriad at middle.
9	Openings of dorsal abdominal scent glands of segments IV and V close to each other.	Openings of dorsal abdominal scent glands of segments IV and V far from each other.	Openings of dorsal abdominal scent glands of segments IV and V rather close to each other.

**Figures 7–14.**
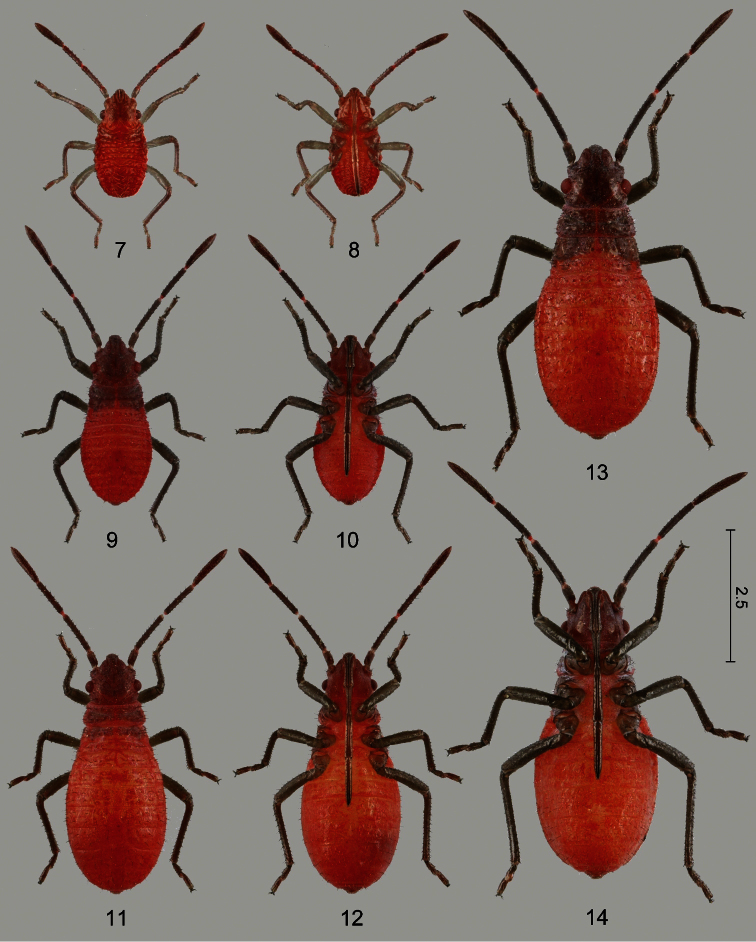
Larvae of *Jadera haematoloma*, dorsal (**7**, **9**, **11**, **13**) and ventral (**8**, **10**, **12**, **14**) views **7–8** first instar (freshly hatched) **9–10** second instar (freshly moulted) **11–12** second instar (old) **13–14** third instar (old). Scale bar in mm.

**Figures 15–20.**
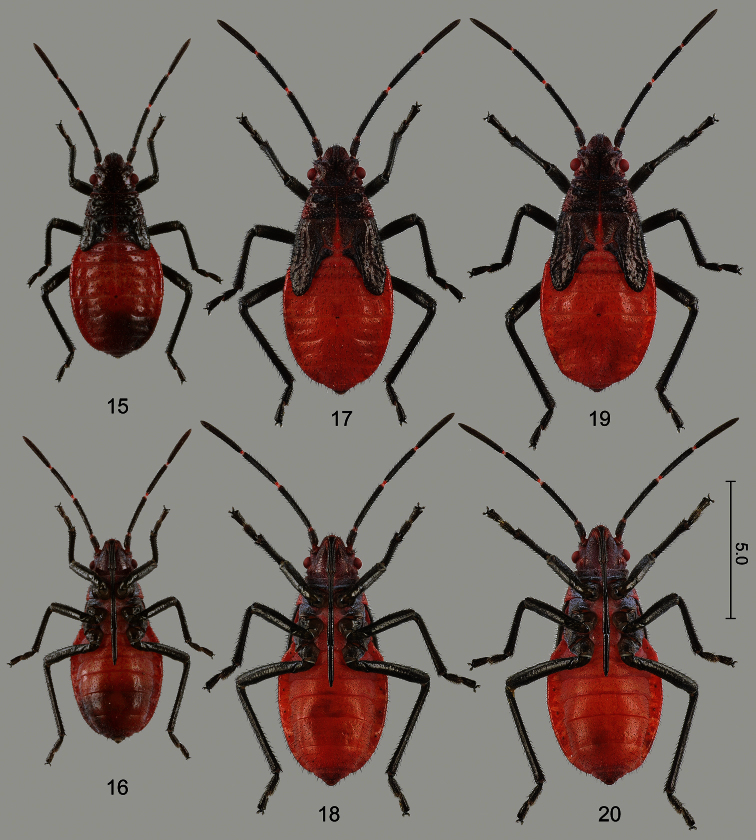
Larvae of *Jadera haematoloma*, dorsal (**15**, **17**, **19**) and ventral (**16**, **18**, **20**) views **15–16** fourth instar (old) **17–18** fifth instar, male (old) **19–20** fifth instar, female (old). Scale bar in mm.

#### Measurements

Provided in Table 6.

**Table 6.** Measurements of larval instars (in mm) and relative lengths of their mesothoracic wing pads collected at site 4. Abbreviations: L1–L5 = 1st–5th larval instars, l = length, w = width.<br/>

**instar**		**body length**^1^	**head length**	**head width**	**head w: l**	**pronotum width**	**pronotum w: head w**	**mesothoracic wing pad^2^**
**L1**	**total**	1.92±0.23<br/> (1.72–2.38)	0.63±0.04<br/> (0.57–0.70)	0.67±0.02<br/> (0.66–0.70)	1.00–1.21	0.75±0.02<br/> (0.74–0.78)	1.09–1.13	absent
	**young** (*N* = 6)	1.72±0.03<br/> (1.72–1.80)	0.59±0.03<br/> (0.57–0.66)	0.68±0.02<br/> (0.66–0.70)	1.00–1.21	0.75±0.02<br/> (0.74–0.78)	1.09–1.13	
	**old** (*N* = 4)	2.17±0.14<br/> (2.09–2.38)	0.67±0.02<br/> (0.66–0.70)	0.67±0.02<br/> (0.66–0.70)	1.00–1.03	0.75±0.02<br/> (0.74–0.78)	1.09–1.13	
**L2**	**total**	3.19±0.38<br/> (2.67–3.73)	0.80±0.04<br/> (0.74–0.82)	0.87±0.03<br/> (0.82–0.90)	1.05–1.17	0.97±0.04<br/> (0.90–1.03)	1.05–1.14	absent
	**young** (*N* = 5)	2.75±0.11<br/> (2.67–2.87)	0.82±0<br/> (0.82–0.82)	0.88±0.02<br/> (0.86–0.90)	1.05–1.10	0.98±0.02<br/> (0.94–0.98)	1.05–1.14	
	**old** (*N* = 10)	3.41±0.23<br/> (3.08–3.73)	0.79±0.04<br/> (0.74–0.80)	0.87±0.03<br/> (0.82–0.90)	1.05–1.17	0.96±0.04<br/> (0.90–1.03)	1.05–1.14	
**L3**	**total**	4.51±0.51<br/> (3.63–5.48)	0.94±0.04<br/> (0.86–0.99)	1.15±0.04<br/> (1.06–1.25)	1.13–1.38	1.24±0.08<br/> (1.06–1.45)	1.00–1.19	minute primordia
	**young** (*N* = 16) 2	3.94±0.30<br/> (3.63–4.49)	0.94±0.04<br/> (0.86–0.99)	1.14±0.04<br/> (1.12–1.22)	1.13–1.29	1.22±0.08<br/> (1.12–1.39)	1.00–1.19	
	**old** (*N* = 18)	4.83±0.25<br/> (4.36–5.48)	0.94±0.05<br/> (0.86–0.99)	1.15±0.05<br/> (1.06–1.25)	1.13–1.38	1.27±0.09<br/> (1.06–1.45)	1.00–1.17	
**L4**	**total**	5.92±0.79<br/> (4.56–7.36)	1.14±0.07<br/> (1.04–1.28)	1.49±0.07<br/> (1.36–1.68)	1.20–1.46	1.66±0.11<br/> (1.44–1.92)	1.03–1.21	I-P to II-M
	**young** (*N*= 10) 2	4.96±0.36<br/> (4.56–5.68)	1.10±0.06<br/> (1.04–1.20)	1.47±0.07<br/> (1.36–1.60)	1.20–1.46	1.61±0.08<br/> (1.44–1.68)	1.03–1.21	I-P to II-M
	**old** (*N* = 19)	6.37±0.45<br/> (5.44–7.36)	1.15±0.06<br/> (1.04–1.28)	1.49±0.07<br/> (1.40–1.68)	1.20–1.46	1.68±0.12<br/> (1.48–1.92)	1.05–1.17	I-P
**L5**	**total**	8.10±1.06<br/> (6.05–10.23)	1.25±0.12<br/> (1.1–1.43)	1.87±0.09<br/> (1.76–2.04)	1.33–1.64	2.32±0.12<br/> (2.20–2.53)	1.18–1.29	II-P to IV-P
	**young** (*N* = 9) 2	6.97±0.78<br/> (6.05–8.14)	1.36±0.08<br/> (1.21–1.43)	1.89±0.10<br/> (1.76–2.04)	1.33–1.55	2.35±0.12<br/> (2.20–2.53)	1.18–1.29	III-P to IV-P
	**old** (*N* = 19)	8.58±0.75<br/> (7.37–10.23)	1.20±0.10<br/> (1.1–1.43)	1.86±0.08<br/> (1.76–1.98)	1.38–1.64	2.31±0.11<br/> (2.20–2.53)	1.18–1.29	II-P to III-P

^1^ 6 individuals of young 3rd, 1 individual of young 4th, and 1 individual of young 5th instars were excluded from measuring the body length because of their shrunken abdomen.<br/>

^2^ The position of the apex of the metathoracic wing pad in respect to the abdominal tergites is given; Roman numerals refer to the segmental homology; A = anterior half; P = posterior half.

#### Morphometric changes during larval development

The body is short and oval in newly hatched larvae (Figs 7–8). Abdomen of older first instar larvae is considerably extended because of feeding, the body therefore more elongate; shape of older larvae gradually becoming more similar to that of adult (Figs 9–20). Second to fifth instar larvae undergo in rather conspicuous changes during each developmental stage (cf. Tables 6–7): larvae of each instar soon after moulting are brighter red, the body appearing smaller because of the shorter abdomen; therefore, the labium is apparently longer in relation to the abdominal sternites. After moulting, the body appears less bright (a dust-like substance on the thorax makes it appear pruinose) and the abdomen extends so it becomes longer and the labium appears relatively shorter when compared to the body length. These changes are demonstrated in two specimens of second instar larvae in Figs 9–10 (freshly moulted) and 11–12 (older). Because of the extension of the abdomen, a small difference can be observed in the relative length of the mesothoracic wing pads of the fourth and fifth instars.

**Table 7.** Variation of the relative length of the labium in different instar larvae (numbers of examined individuals). The position of the apex of the labium in respect to the abdominal sternites is given; Roman numerals refer to the segmental homology; A = anterior half; P = posterior half.<br/>

	**II**	**III**		**IV**		**V**		**VI**		**VII**	**VIII**	**IX**	**X**	**XI**
	**P**	**A**	**P**	**A**	**P**	**A**	**P**	**A**	**P**	**P**				
**L1 (newly hatched)** (*N*= 6)														6
**L1 (old)** (*N*= 4)					1		3							
**L2 (young)** (*N* = 5)										5				
**L2 (old)** (*N* = 10)				1	4	2	3							
**L3 (young)** (*N* = 10)						4	4	1	1					
**L3 (old)** (*N* = 18)			3	7	3	4	1							
**L4 (young)** (*N* = 7)					1	1	3	1	1					
**L4 (old)** (*N* = 19)		6	4	7	2									
**L5 (young)** (*N* = 9)			1	2	2	3	1							
**L5 (old)** (*N* = 19)	5	8	4	2										

#### Host plants and feeding

Several adults and larvae were observed feeding on the ripe fruits of *Cardiospermum halicacabum* (sites 1–4) and *Koelreuteria elegans* subsp. *formosana* (sites 5–7).

At sites 1, 4, 5, 6 and 7, the thick layer of dead leaves accumulated below the host plant offered an ideal microhabitat for adults and larvae. First and second larval instars were never observed on the plants; they hid among the leaf litter (Fig. 27) and fed mostly on fallen, mature fruits (with brownish pericarp), which were open (Fig. 30). At site 4 most of the first to third instar larvae aggregated within the ripe and open fruits. Third instar and older larvae were more vagile than the first two instars; they walked around and frequently climbed and formed aggregations on the stem of the heartseed vine, on the trunk of *Koelreuteria elegans* subsp. *formosana* (commonly hiding in the crevices), and occasionally on the shadow side of cement pillars around the plants.

**Figures 21–27.**
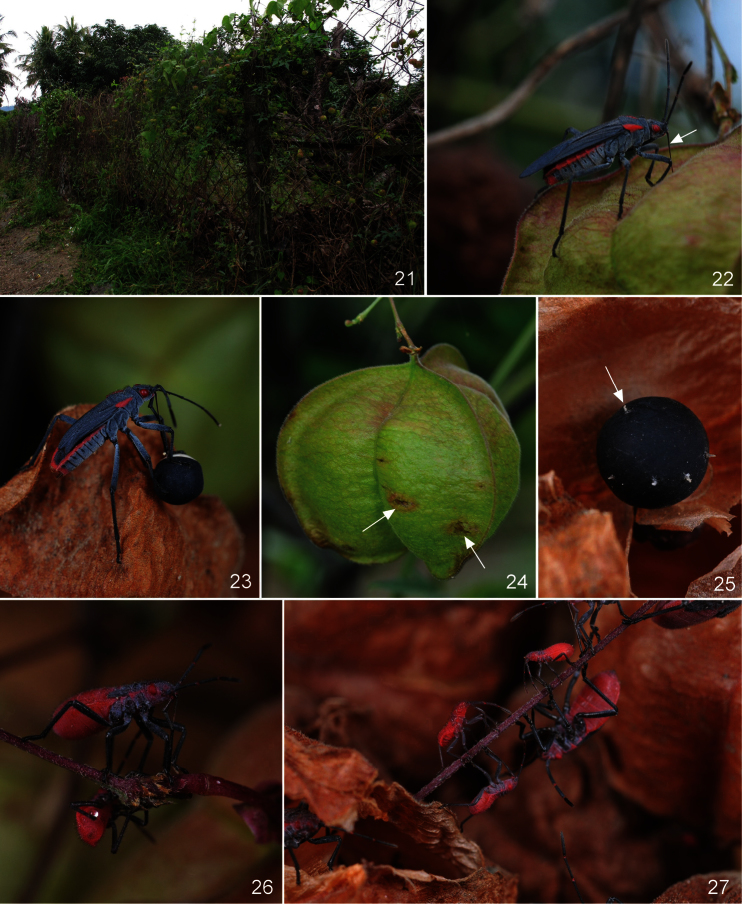
*Jadera haematoloma* on and around its host plant (*Cardiospermum halicacabum*) (site 1). **21** Chain-link fence with heartseed vine **22** Adult male feeding on a fruit (arrow: stylet with its basal portion ensheathed in the concavity of labrum) **23** Adult male feeding on a seed **24** fruit damaged by *Jadera haematoloma* (arrows: feeding scars) **25** seed damaged by *Jadera haematoloma* (arrow: feeding cone) **26** fourth instar larva feeding on the stalk of a fruit **27** larvae walking and feeding on a leaf stem of heartseed vine among leaf litter.

**Figures 28–34.**
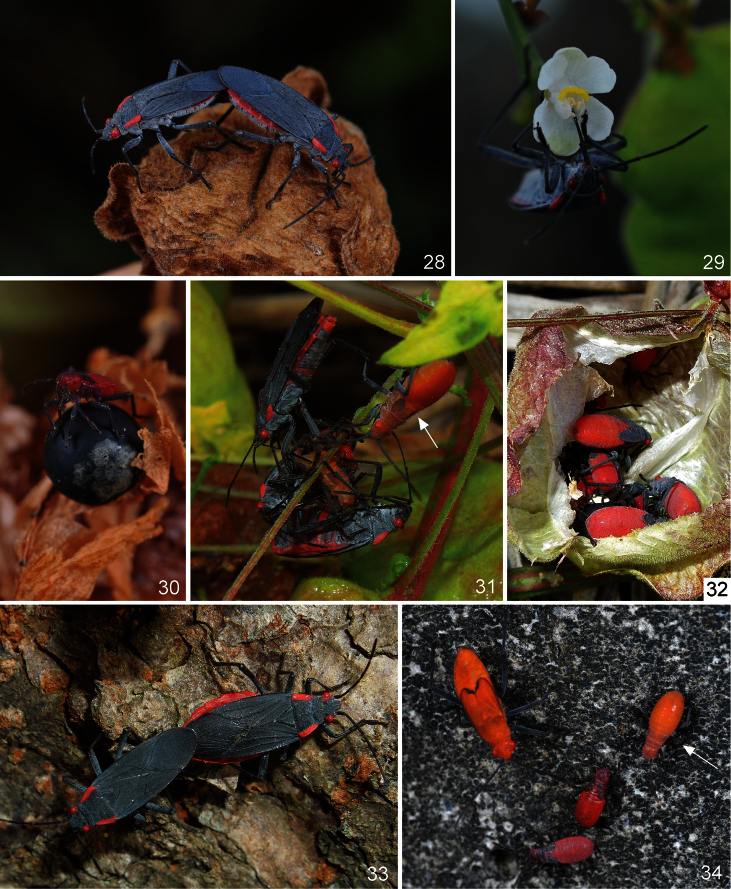
*Jadera haematoloma* on and around its host plant (*Cardiospermum halicacabum*). **28** A mating couple, the female (in the right) feeding on fruit of the host plant **29** Adult feeding on a flower of *Cardiospermum halicacabum* **30** second instar larva feeding on seed of the host plant **31** Adults feeding on a fourth instar larva of *Leptocoris augur* (arrow: another fourth instar larva of *Leptocoris augur*) **32** fifth instar larvae in the fruit of *Cardiospermum halicacabum* **33** a male guarding a gravid female **34** aggregation of a brachypterous male, a fourth instar larva (arrow) of *Leptocoris augur* and two fourth instar larvae of *Jadera haematoloma*.

During feeding, stylets of adults and fourth and fifth instar larvae penetrated deeply into fruit through the pericarp (Figs 22: arrow, 28), and reached the seeds. Brown spots appeared on fruits where it was damaged by the feeding of adults or older larvae (Fig. 24). All larval instars accessed seeds by climbing into the fruit through an opening or injury to the pericarp (Fig. 32), or consumed fallen seeds; several adults also fed similarly. Adults and all (including first) larval instars were frequently sucked the fruit stalks (Fig. 26). Adults and at least older larvae commonly drank nectar from flowers of *Cardiospermum halicacabum* (Fig. 29) too. Frequent nectar consumption from flowers of *Bidens pilosa* var. *radiata*, an asteracean weed, was observed at site 8 (U. Ong, *pers. comm*.).

As in several other Pentatomomorpha, especially those consuming seeds or feeding from other hard surfaces, the feeding is of the stylet-sheath type (Miles 1972, Cobben 1978). The feeding cones formed by solidified saliva on a seed of *Cardiospermum halicacabum* are shown in Fig. 25 (arrow). Feeding cones also were observed in the case of specimens preying on other arthropods (Fig. 36: arrow), but they never appeared when the specimens fed on the fruit stalks.

**Figures 35–36.**
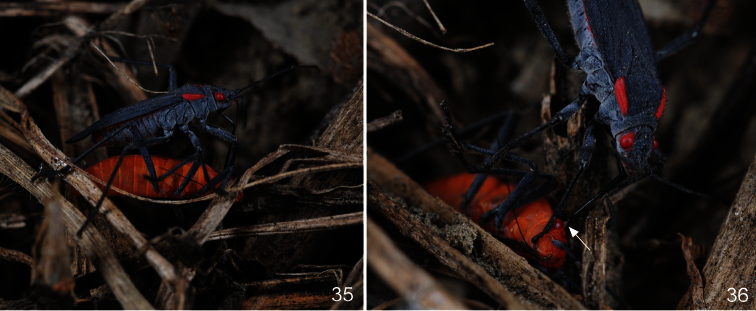
A female of *Jadera haematoloma* attacking a female of *Leptocoris augur* (site 1). In Fig. **36** arrow shows the feeding cone.

Dozens of specimens were kept for several days in captivity on seeds of *Cardiospermum halicacabum*, but no cannibalism was observed. Nevertheless, several instances of zoophagy on other rhopalid species were observed; these are discussed below under ‘Competitors’.

#### Condition of populations in Taiwan

At some places the abundance of *Jadera haematoloma* was rather high on and around its host plant. At site 1 an hour of searching along the fence (in an area of about 10 × 1 m, cf. Fig. 21) yielded 43 adults and dozens of larvae. A few minutes of searching resulted in hundreds of larvae at site 4; aggregations composed by about 10–30 specimens were observed at this locality.

In the time of the observations (between 6.xi.2012 and 14.i.2013) specimens were still walking, running and feeding actively, and did not show any sign of diapause. Several mating pairs were found (6 at site 1, 2 at site 3, 5 at site 4, 6 at site 7, several at site 8) (Fig. 28). Many of the copulating females were gravid, with greatly enlarged abdomens (Fig. 33); a large number of gravid females was found on *Koelreuteria elegans* subsp. *formosana* during December 2012 and January 2013.

Several dozen larvae were observed and approximately one hundred were captured. At site 1 (30.ix) only first to third instars were found; careful searching did not yield any older instars. All larval instars were observed at site 4 on the same day, forming aggregations of dozens of larvae at ground level.

The largest number of adults was collected at site 1 (30.xi.2012) and at site 5 (3.xii.2012 and 14.i.2013). Estimating from this limited sample, all of these populations were apparently distinctly male-biased, with a ratio of 1.75:1 (28 ♂♂, 16 ♀♀) in the first case, 2.4:1 (38 ♂♂, 16 ♀♀) in the second case, and 3:1 (42 ♂♂, 14 ♀♀) in the third case.

#### Competitors

In all localities *Jadera haematoloma* co-occurred with *Leptocoris augur* (Fabricius, 1781), a taxonomically closely related serinethine species native and rather abundant in Taiwan. In all of the above localities *Leptocoris augur* was estimated to be clearly more abundant than *Jadera haematoloma*. Individuals of both species frequently occur within the same aggregation (Fig. 34).

According to our subjective observations, adults and especially larvae of *Jadera haematoloma* are more vagile than *Leptocoris augur*. Although first and second instars usually do not walk much, they ran quickly when approached, and they were distinctly quicker than larvae of *Leptocoris augur* of the same instar. The difference in older instars also was evident.

*Leptocoris augur* was observed to feed on both *Cardiospermum halicacabum* and *Koelreuteria elegans* subsp. *formosana* in a manner similar to that described above for *Jadera haematoloma*; nectar feeding in *Leptocoris augur* also was observed. In some cases direct interference between individuals of the two species was observed. At site 2, adults and larvae were observed to feed on a freshly moulted adult of *Leptocoris augur* (Fig. 31). A similar phenomenon was observed in a plastic container where the two species were reared together: a freshly moulted adult of *Leptocoris augur* was attacked and consumed by six larvae (representing all larval instars) of *Jadera haematoloma*. At site 1 a female of *Jadera haematoloma* was observed to approach a female of *Leptocoris augur*, climb its back, and penetrate its labium into its neck (Figs 35–36). They remained in this position for about 15 minutes; after that the individual of *Leptocoris augur* was still alive but it stopped moving and died a few hours later.

At site 5 another related native rhopalid having similar host plants and habits, *Leptocoris vicinus* (Dallas, 1852) was observed. Adults and all larval instars of *Leptocoris vicinus* were found on the ground, but in smaller numbers than *Jadera haematoloma* and *Leptocoris augur*.

## Discussion

Our field observations indicate that *Jadera haematoloma* has probably already established in southern Taiwan. Because of the large number of adults, high frequency of mating pairs, presence of several gravid females, and most importantly the large numbers of all larval instars, it is apparent that strong, reproducing populations are present in southern Taiwan. The number and the condition of the observed populations suggest that *Jadera haematoloma* was not introduced in 2012, but at least one or two years earlier. From the current geographic distribution within Taiwan it seems probable that the species entered through the seaport of Kaohsiung, the largest harbour of the country where most of Taiwan’s marine import and export passes.

Apparently the populations in Taiwan have a host range similar to those in North America. *Cardiospermum halicacabum* and *Koelreuteria elegans* subsp. *formosana* were identified as host plants of *Jadera haematoloma* in Taiwan; both plants previously were reported as hosts in the continental USA, the Caribbean and Hawaii (Table 1). Frequent nectar consumption from host flowers and Bidens *pilosa* var. *radiata* was observed.

Little is known about the bug’s phenology in Taiwan. Active, reproducing populations fed on both *Cardiospermum halicacabum* and *Koelreuteria elegans* subsp. *formosana* from late November to mid-January. Because of the subtropical and tropical climate of Taiwan, no winter diapause is expected. Because fruits of balloon vine are available year round in Taiwan, and seeds of *Koelreuteria elegans* subsp. *formosana* also are available until late March (Chou and Chen 2010), the population presumably will not enter starvation diapause but remain active, at least on *Cardiospermum halicacabum*. Further field observations are needed to confirm or reject this hypothesis.

At least several populations in Taiwan seem more or less male-biased and show variation similar to those in the southern USA. Females are significantly larger than males in both wing morphs and macropterous morphs are significantly larger than brachypters, which is similar to the North American populations (Carroll et al. 2003b). Carroll et al. (1997, 1998, 2001) reported slight differences in the body size of populations feeding on different host plants (*Cardiospermum corindum*, *Koelreuteria elegans*) in Florida; no difference in body size was found in populations feeding on *Cardiospermum halicacabum* and *Koelreuteria elegans* subsp. *formosana* in Taiwan.

11.4% of the individuals in the population at site 1 observed on 30.xi.2012 were brachypterous; this ratio is about 20% in the southern USA (Carroll et al. 2003b). No inference can be drawn from this apparent difference because our observations are based on a much smaller sample. In some populations on *Cardiospermum halicacabum* (site 8, 13.i.2013) the majority of the specimens occasionally is brachypterous. In spite of considerable effort and observations at several localities no brachypterous individuals were observed on *Koelreuteria elegans* subsp. *formosana*. We suggest that wing polymorphism can be explained as a response to host-plant phenology: the percentage of the brachypterous specimens is higher on *Cardiospermum halicacabum*, which produces seeds year round, but brachypters are rare or absent on *Koelreuteria elegans* subsp. *formosana*, which is highly seasonal with respect to seed production. A similar negative correlation between food availability and macroptery ratio was demonstrated in laboratory experiments by Dingle and Winchell (1997).

*Jadera haematoloma* occurs in the same habitats and uses the same food in the same manner as do *Leptocoris augur* and *Leptocoris vicinus*, two taxonomically closely related native rhopalid species in Taiwan. Mixed-species aggregations of *Jadera haematoloma* and one or both of the native species were commonly observed at several localities. Although no interspecific competition between *Jadera haematoloma* and other hemipterans was reported in North America (Carroll 1988), at least scramble competition with the two *Leptocoris* species is expected if resources are limited. We observed direct interference between individuals of *Jadera haematoloma* and *Leptocoris augur*; based on our preliminary observations *Jadera haematoloma* is usually more successful in such interferences. Although *Jadera haematoloma* also readily feeds on various disabled or freshly dead arthropods in its native area (Carroll and Loye 1987, Ribeiro 1989), feeding on *Leptocoris augur* seems particularly common in Taiwan. Further investigation is needed on the biological interaction between *Jadera haematoloma* and the two native rhopalid species and its effect on their populations.

*Koelreuteria elegans* subsp. *formosana* originally was found mainly at lower altitudes (Chen 1993), but during the past few decades it became a popular ornamental tree planted extensively in Taiwan along roads in major cities and in the country (Chang 2005, Chen 2006). *Cardiospermum halicacabum* is common throughout the main island of Taiwan and occurs in various habitats, most frequently along the coast and in wastelands and fallows. An additional 7 genera of Sapindaceae, each with a single species, have been recorded from Taiwan (Chen 1993). Among them, *Sapindus mukorossii* was listed as a host plant of *Jadera haematoloma* in the USA (Carroll and Loye 2012). *Allophylus timorensis* Blume, an evergreen shrub common in thickets along the coast of southern Taiwan (Chen 1993), is also a likely host plant because adults and larvae of *Leptocoris vicinus* frequently feed on its seeds in large numbers in the Pratas Islands and the main island of Taiwan (J.F. Tsai, *pers. observ*.). Colonization of further sapindacean species also seems likely. Because the tropical and subtropical climate of Taiwan is suitable for the species and several of its host plants are readily available, further rapid spread is expected. *Jadera haematoloma* probably will colonize all of the main island of Taiwan. A specimen from central Taiwan (Chiayi County) apparently indicates that such spread is in progress.

Several of the sapindacean plants that have already been reported as host plants of *Jadera haematoloma* in the USA also occur in southeast China (Lo and Chen 1985). *Cardiospermum halicacabum* is of circumtropical distribution and is common in the eastern, southern and western parts of China; it also occurs in the northern and northeastern parts of the country but is more rare. The genus *Koelreuteria* is represented by *Koelreuteria paniculata* and *Koelreuteria bipinnata* in China, both of which have been reported as hosts of *Jadera haematoloma* in the USA (Table 1). *Sapindus mukorossii* and three additional congeners are widely distributed in eastern, southern and western China. Several other members of the rich sapidancean flora of China, comprising 25 genera and 53 species (Lo and Chen 1985), could potentially be consumed by *Jadera haematoloma* in case of an eventual invasion. Because the climate of a great part of Southeast Asia and even the neighbouring Palaearctic areas are presumably suitable for *Jadera haematoloma*, and various host plants occur in the region, an eventual introduction might also result in establishment of the species in other regions of Southeast Asia.
